# The CDC blood lead reference value for children: time for a change

**DOI:** 10.1186/s12940-019-0457-7

**Published:** 2019-02-28

**Authors:** Jerome A. Paulson, Mary Jean Brown

**Affiliations:** 1 George Washington University School of Medicine & Health Sciences, and George Washington University Milken Institute School of Public Health, 1113 N Howard St, Alexandria, VA 22304-1627 USA; 2 Harvard TH Chan School of Public Health, 665 Huntington Ave, Boston, MA 02115 USA

**Keywords:** Blood lead levels, Primary prevention

## Abstract

The purpose of this article is to consider alternate uses of the blood lead reference value for children. There are two possible approaches. Historically the reference value has been used to guide clinical and public interventions for individual children. As the distribution of blood lead levels in the population has been lowered over time, the blood lead level at which interventions are recommended has also been reduced. The use of a reference value of 3.5 μg/dL, based on the 98 percentile of blood lead levels for children in 2011–2014 National Health and Nutrition Examination Survey is under review. For several reasons, adopting the new reference value to guide clinical and public health management puts practitioners in an untenable position. First, the changes in the brain caused by lead are significant and persistent. However, these adverse impacts are subtle and although clearly identified at the population level, not predictive for individual children. In addition, the recommended interventions have not been shown to reduce blood lead levels once they are elevated. Finally, clinical laboratory and office-based blood lead testing devices are not required to quantify blood lead levels < 4 μg/dL and in many cases cannot reliably test for low blood lead levels. Revising the reference value also will undoubtedly result in diversion of resources away from those population-based interventions which have demonstrated success. We argue for second approach, in the management of lead poisoning in the US from one of evaluation and management at the individual level to one of population based primary prevention. This would require a strategy directed at controlling or eliminating lead in children’s environment before they are exposed. The reference value, as a benchmark, is essential to ensure that primary prevention efforts are successful.

## Background

Lead is known to cause neurocognitive problems in children; and there is no known blood lead threshold below which injury does not occur [1]. Reducing blood lead levels (BLLs) to less than 2 μg/dL among all US children born in 2018 would result in an overall savings of approximately $23.5 billion over the lifetimes of these children and children born in the subsequent 5–10 years could expect to reap similar benefits [2]. These savings are the result of small savings in health care, primarily Medicaid, and somewhat larger savings for educational support and juvenile justice costs for affected children but are primarily the result of the expected productivity increases for children who will be able to achieve their potential.

Historically the approach used to protect children from the adverse health effects associated with lead exposure has been for the Centers for Disease Control and Prevention (CDC) to lower the BLL at which individualized clinical and public health interventions are recommended as data describing adverse effects of lead at lower and lower BLLs became available. In 2012 CDC adopted a reference value, for BLL in children rather than defining a ‘level of concern’ or ‘toxicity’. A reference value is a statistic derived from the distribution of the concentration of a specific compound or element in blood or other body fluid of a reference [3]. Reference values are particularly valuable to characterize individual results as “elevated” or “not elevated” in comparison to the population mean value for chemicals for which there is no known safe concentration. The 97.5 percentile of the distribution is often used to define “elevated”. The NHANES data provide an appropriate source for characterizing a reference value for BLLs in children 1–5 years old [4]. In 2012 this value was 5 μg/dL. The CDC subcommittee on lead poisoning prevention has recently recommended to CDC that the reference value for blood lead in children be revised to 3.5 μg/dL, based on the 2011–2014 National Health and Nutrition Survey (NHANES) (National Center for Environmental Health Board of Scientific Counselors, 2016). How this new reference value will be implemented in practice remains under discussion. Below we offer recommendations for the future use of the BLL reference value.

### Diagnosis and treatment

The use of the reference value to establish a threshold of individualized intervention is fraught with problems. First, most clinical and office based BLL testing methodologies lack precision in measuring BLLs at value < 4 μg/dL. In a recent review of 5 years of blood lead proficiency testing, clinical laboratories participating in CDC’s Lead and Multi-Element Proficiency quality assurance program (LAMP), 40% of participating laboratories were unable to quantify blood lead levels of 1.48 μg/dL in reference samples; and 23% reported limits of detection between 3 and 5 μg/dL) [5]. In addition, the Comprehensive Laboratory Improvement Act (CLIA) current criteria for accuracy for blood lead values < 40 μg/dL is a range of +/− 4 μg/dL. Thus, the true value of a blood measurement of 4 μg/dL can range from 0 to 8 μg/dL. As a result, measuring BLLs at or near the revised reference value is an analytical challenge and one that cannot currently be met in the most cases.

Second, the recommended interventions for children with BLLs 5–44 μg/dL include home visits and environmental inspections, case management and control of environmental lead sources. Although these services have been demonstrated to lower environmental lead levels, they are not successful in reducing BLLs in children who are already elevated [6].

Third, the behavioral and academic outcomes for any individual are often subtle and difficult to predict [1]. Studies that have examined the association between the rate of natural reductions in BLLs and neurodevelopment have found that deficits related to early exposure are persistent. Therefore, the only certain way to avoid lead-associated neurodevelopmental morbidity is to prevent exposure in the first place—primary prevention remains the best course of action [7].

### Primary prevention

Our primary prevention strategies have been far more successful. In the US, we have been successful in lowering BLLs in cohorts of children born after regulations were implemented to reduce various sources of lead [4]. Between 1978 and 2012 BLLs in children were reduced by 93% as regulations requiring the elimination of lead in gasoline, decreased water lead levels and control or elimination of lead in soil, house dust, paint and consumer products, were promulgated. Thus, sparing millions of children from the very high BLLS once prevalent in the US [7].

This success is incomplete because the benefits have not been realized uniformly across communities and there remain areas where we know that the risk for exposure is disproportionately high, particularly areas with high concentrations of pre-1950s housing, those with legacy contamination from former industrial use, and in certain sub-populations where we know cultural practices put children at risk of exposure to lead in traditional products and medicine [8, 9].

## Conclusion

Adopting the latest reference value as a threshold for individualized interventions places pediatric and public health practitioners in an untenable position. Given the limitations in laboratory diagnosis, the lack of effective interventions and the inability to predict outcomes at the individual level, the practice of waiting for a child’s BLL to rise to a predetermined ‘reference value’ or ‘level of concern’ before reducing environmental lead contamination has outlived its usefulness. We recommend instead a shift in the US to primary prevention focused on controlling or eliminating lead in children’s environment before they are exposed. Nonetheless the reference value for BLL will continue to be crucial as a benchmark for tracking whether our efforts to control or eliminate these sources are successful.

To eliminate lead’s effect on children’s futures, we must focus our efforts to prevent children from being exposed to lead in those areas at highest risk before exposure occurs while maintaining resources to respond to quickly when children have been exposed. Children, families and society achieve the most benefit from interventions that ensure that all babies come home to a lead safe environment.

At the national level, the President’s Task Force on Environmental Health Risks and Safety Risks to Children was recently charged with the development and implementation of a new Federal Strategy to Reduce Childhood Lead Exposures and Eliminate Associated Health Impacts. Lead Task Force agencies include the Department of Health & Human Services including CDC, the Center for Medicaid Services and the Maternal Child Health Bureau, the Environmental Protection Agency and the Department of Housing and Urban Development’s Office of Lead Hazard Control and Healthy Homes. We would like to see this taskforce expanded to include representatives of local and state public health and housing agencies, pediatric health care providers, child health advocates and parents to provide diverse and “on the ground” perspectives. The recently reconstituted federal advisory committee on Lead Exposure and Prevention offers another avenue for a robust, inclusive discussion of lead poisoning prevention strategies appropriate for the twenty-first Century.

Communities can be key players in reducing childhood lead exposures. With sufficient resources communities can identify all residential buildings that may be at risk of exposing a child to lead. For example, a first step would be to require the comprehensive inspection and elimination of the lead hazards in all units in a building where one child with a high blood lead level has been identified. Subsequently, a more comprehensive program of building identification and renovation can be developed and implemented. In most communities, the environment will be made ‘lead safe’ rather than lead free. Thus, routine maintenance of buildings, soil coverings and water treatment will be necessary until these sources of lead are completely removed from children’s lives.

The US can no longer use children to identify lead hazards in the community, rather than waiting for children to meet an arbitrary BLL threshold, we need to muster the resources, and resolve needed to prevent exposure to lead before it occurs. We know what to do. The evidence of the last 40 years demonstrates that we can prevent high blood lead levels in children by controlling or eliminating lead sources in their environments. Forcing the statistical construct of a reference value to act as a clinical intervention level does not get us there.

## Abbreviations

BLL: Blood lead level
CDC: US Centers for Disease Control and Prevention
CLIA: Clinical Laboratory Improvement Act
NHANES: National Health and Nutrition Examination Survey
US: United States
